# Pigment Epithelium-Derived Factor and Sex Hormone-Responsive Cancers

**DOI:** 10.3390/cancers12113483

**Published:** 2020-11-23

**Authors:** Naomi Brook, Emily Brook, Crispin R. Dass, Arlene Chan, Arun Dharmarajan

**Affiliations:** 1 School of Pharmacy and Biomedical Science, Curtin University, Bentley, WA 6102, Australia; naomi.brook@postgrad.curtin.edu.au (N.B.); emily.brook@postgrad.curtin.edu.au (E.B.); 2 Curtin Health Innovation Research Institute, Bentley, WA 6102, Australia; 3 School of Medicine, Curtin University, Bentley, WA 6102, Australia; Arlene.Chan@curtin.edu.au; 4 Breast Cancer Research Centre-Western Australia, Hollywood Private Hospital, Nedlands, WA 6009, Australia; 5 Department of Biomedical Sciences, Sri Ramachandra Institute of Higher Education and Research, Chennai 600116, India

**Keywords:** pigment epithelium-derived factor, oestrogen, androgen, cancer, metastasis

## Abstract

**Simple Summary:**

The ongoing clinical need to improve cancer therapies warrants a better understanding of the mechanisms behind cancer growth and its spread to distant organs. To this end, research into the impact of sex hormones, such as oestrogens and testosterones, in cancers has led to improvements in the way sex hormone-responsive cancers are treated. Pigment epithelium-derived factor (PEDF) is a protein with anti-cancer properties that is also sensitive to sex hormones. The aim of this review is to explore what is currently known about sex hormones and PEDF in cancers in order to better understand the anti-cancer role of PEDF in sex hormone-responsive cancers.

**Abstract:**

Oestrogens and androgens play important roles in normal and cancerous tissue and have been shown to negatively regulate pigment epithelium-derived factor (PEDF) expression in sex hormone-responsive tumours. PEDF suppresses tumour growth and its downregulation by oestrogen is implicated in tumorigenesis, metastasis, and progression. PEDF expression is reduced in cancerous tissue of the prostate, breast, ovary, and endometrium compared to their normal tissue counterparts, with a link between PEDF downregulation and sex hormone signalling observed in pre-clinical studies. PEDF reduces growth and metastasis of tumour cells by promoting apoptosis, inhibiting angiogenesis, increasing adhesion, and reducing migration. PEDF may also prevent treatment resistance in some cancers by downregulating oestrogen receptor signalling. By interacting with components of the tumour microenvironment, PEDF counteracts the proliferative and immunosuppressive effects of oestrogens, to ultimately reduce tumorigenesis and metastasis. In this review, we focus on sex hormone regulation of PEDF’s anti-tumour action in sex hormone-responsive tumours.

## 1. Introduction

Sex hormones including oestrogens, progesterone, and androgens play important roles in regulating development and physiological function [1,2,3]. Conversely, aberrant sex hormone signalling is implicated in tumorigenesis, metastasis, progression, and treatment resistance in cancers arising in sex hormone-responsive tissues [4].

## 2. Sex Hormones

Oestradiol (E2; (17β)-estra-1,3,5(10)-triene-3,17-diol) is the major circulating bioactive oestrogen in premenopausal women, and oestrone (E1; 3-hydroxyestra-1,3,5(10)-trien-17-one) is the main bioactive oestrogen in postmenopausal women [5]. E1 is reversibly converted to E2 and to oestrone sulphate (E1S; estra-1,3,5(10)-trien-17-one 3-sulfate), a non-active oestrogen stored in plasma and tissues. During menopause, circulating E2 levels decrease as ovarian steroidogenesis diminishes [5]. Whilst increased aromatisation of E1 and E2 occurs in adipose and breast tissue. The latter also occurs in liver, bone, and brain—these being frequent sites for metastases in BC. E1S is the most abundant circulating oestrogen regardless of menopausal status and is an important E1 precursor in post-menopausal women [6]. Despite low levels of circulating E2, intratumoral E2 is higher in malignant oestrogen receptor positive (ER+) BC tissue compared to normal breast tissue in postmenopausal patients [7]. It is not known whether systemic oestrogen uptake or local synthesis accounts for this paradox. Notwithstanding, future studies should strive to simulate the oestrogenic environment present in pre- and postmenopausal BC patients to ensure that any findings are better aligned to the actual hormonal conditions in patients. In this manner, results may lead to development of future therapies. When considering metastatic BC studies, it is important to take into consideration local production of oestrogens at the site of metastases. For instance, bone has a potent local oestrogen axis, which is highly attractive to circulating osteotropic BC cells [8].

The primary androgen found in men is testosterone (T; androst-4-ene-17β-ol-3-one), which is produced by the Leydig cells of the testes, and released into circulation. The androgen signal can be further amplified in selected target tissues where T is reduced to 5α-dihydrotestosterone (DHT, 5α-androstan-17β-ol-3-one), considered to be the most potent natural androgen [9]. DHT is the 5α-reduced metabolite of testosterone (T) that is principally converted from T in target organs such as prostate, skin, and liver [10]. Synthesis can also occur from other precursors, which is important in the prostate but of lesser importance in other tissues. Intracellular DHT is a more potent androgenic agonist than T, and its presence in some tissues such as the prostate is necessary for organ development and function. Circulating DHT levels are of much less importance than circulating T levels for optimising intracellular DHT concentrations. Inhibition of 5α-reductase diminishes the agonist action of T. It is well documented that DHT can be synthesised in androgen-sensitive tissues such as prostate from substrates other than T (for example, 17-hydroxypregnenolone and 17-hydroxyprogesterone in what is termed the “backdoor” pathway and from 5α-androstane-3α,17-β-diol via the intracrine reverse synthesis pathway) [11]. Castration-resistant PC (CRPC) retains AR activity through a variety of mechanisms, including being able to maintain high androgen levels within tumour cells despite undetectable or low circulating concentrations [12]. In bone metastasis for instance, PC cells can convert adrenal androgens to T and DHT, thereby uncoupling reliance on circulating hormone levels [13].

## 3. Background on Pigment Epithelium-Derived Factor (PEDF)

Pigment epithelium-derived factor (PEDF) is a 50 kDa human glycoprotein encoded by the *SERPINF1* gene on chromosome 17p13.3 [14]. Since its discovery as a neurotrophic factor in the eye, PEDF is now known to support neurogenesis, regulate angiogenesis, mitigate oxidative stress, inhibit inflammation, regulate stem cell niches, promote osteogenesis, and inhibit tumorigenesis and metastasis [15,16,17]. Sex hormones regulate PEDF expression in some tissues. E2, progesterone, and human chorionic gonadotrophin negatively regulate ovarian PEDF expression to coordinate follicular angiogenesis during ovulation [18,19]. In the endometrium, PEDF expression is negatively regulated by E2 and positively regulated by progesterone throughout the secretory phases of the menstrual cycle in premenopausal women [20]. DHT negatively regulates PEDF expression in the prostate to promote normal tissue development [21]. A summary of the regulatory role of sex hormones on PEDF expression, plus the role of PEDF in the original tissue, is provided in Table 1. Sex hormones drive cellular proliferation and tissue expansion, whilst PEDF primarily does the opposite, thus an antagonistic relationship is expected. The remainder of this review will focus on the biological effects of PEDF in tumours arising in sex hormone-responsive tissues, focusing on cancers of the breast, prostate, ovary, endometrium, and cervix. A summary of the anti-tumour effects of PEDF in these tissues, including key pathways and effectors involved, is provided in Table 2.

## 4. PEDF and Cancer

### 4.1. Prostate Cancer

Androgens such as testosterone and androstenedione are sex hormones that bind androgen receptors (ARs) expressed by prostate cells to regulate cell growth [2]. Age-related changes in androgen sensitivity may be involved in the development of prostate cancer (PC) [2]. PEDF expression is reduced in PC tissue compared to normal tissue, with in vivo studies revealing that PEDF elicits direct pro-apoptotic effects on PC cells [21]. Circulating PEDF levels may also be dysregulated in PC. Serum PEDF levels are decreased in patients with higher Gleason grade prostate tumours compared to lower grade tumours, indicating that serum PEDF levels may predict early stage PC [36]. Serum PEDF levels appear to be decreased in PC patients with extracapsular extension compared to those without and inclusion of PEDF in biomarker panels may predict for locally advanced PC [37]. Similarly, decreased serum PEDF in patients with benign prostate hyperplasia and PC compared to healthy individuals has also been reported [38]. Elevated plasma PEDF levels positively correlated with tumour grade in PC patients [39]. Loss of PEDF expression in PC tissue may be associated with a more metastatic phenotype, with PEDF expression inversely proportional to tumour grade and MVD in prostate tumours [40]. Body mass index effects circulating PEDF levels [41], so it is likely that obesity will also impact circulating and tissue PEDF levels in patients with sex hormone-responsive tumours. These studies indicate that loss of PEDF in PC is permissive for tumour growth, metastasis and progression.

PEDF may inhibit PC growth and metastasis by downregulating transforming growth factor beta (TGF-β), signal transducer and activator of transcription 3 (STAT3), and androgen receptor (AR) signalling in order to control the expression of genes involved in apoptosis, cell cycle regulation, metastasis, and invasiveness in PC cells [42]. PEDF appears to mediate pro-apoptotic effects by directly upregulating caspase activity in PC cells, including caspase-3 and caspase-7 [32]. PEDF directly promotes PC cell apoptosis by triggering cell death signalling pathways and subsequent activation of caspases [31]. This study found that PEDF activates cell death pathways in PC cells by interacting with the laminin receptor (LR), which then activates JNK/PPAR-γ signalling to upregulate Fas ligand (FasL) and promote casapase-mediated apoptosis. PEDF also appears to reduce PC cell tumorigenic and metastatic potential by upregulating PAI-2, which prevents activation of urokinase plasminogen (uPA) and other proteases involved in metastasis [28]. PEDF is also able to increase PC cell sensitivity to chemotherapy by upregulating Il-8/C-X-C motif receptor 1 (CXCR1) signalling and reducing PC cell proliferation, with potential to reduce the occurrence and burden of clinical PC metastases [34]. PEDF may improve clinical responses to chemotherapy by enhancing macrophage-mediated phagocytosis of PC cells and by reducing PC cell invasiveness [33]. These findings indicate that PEDF blocks key biological steps essential for tumorigenesis and metastasis and may enhance the cytotoxicity of established anti-cancer treatments by increasing anti-tumour immune responses.

**Figure 1:**
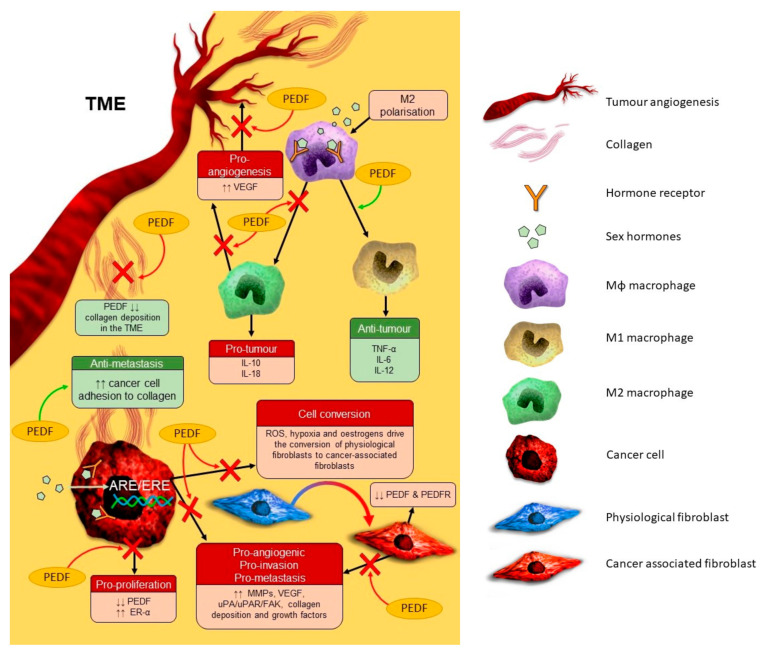
The complex interplay between sex hormones and pigment epithelium-derived factor (PEDF) in tumorigenesis and metastasis. This figure focuses on the inhibitory effects of PEDF. Key: ARE, androgen response element; ER, oestrogen receptor; ERE, oestrogen response element; FAK, focal adhesion kinase; Il, interleukin; M2, type of macrophage; MMP, matrix metalloproteinase; PEDFR, PEDF receptor; ROS, reactive oxygen species,; TME, tumour microenvironment; TNF, tumour necrosis factor; uPA, urokinase plasminogen activator; uPAR, uPA receptor; VEGF, vascular endothelial growth factor; ↑↑, increased; ↓↓, decreased. Red arrows and crosses indicate pathways blocked by PEDF; green arrows indicate pathways activated by PEDF. Red fields represent pro-tumour mechanisms and green fields represent anti-tumour mechanisms.

The tumour microenvironment (TME) is a key driver of tumorigenesis and tumour-resident immune cells secrete pro-inflammatory mediators, such as macrophage-derived TNF-α, to downregulate PEDF and promote prostate tumour growth, angiogenesis, and metastasis [43]. PEDF appears to regulate phagocytic signalling cascades in the prostate TME to enhance macrophage recruitment, M1 polarisation, and macrophage tumoricidal action [35] (Figure 1). Specifically, PEDF appears to downregulate anti-phagocytic CD-47 and upregulate pro-phagocytic PEDF receptors PEDF-R (ATGL/PNPLA2) and ATP synthase F1 subunit beta (ATP5B) on macrophages, which then promotes macrophage-mediated tumoricidal action on PC cells via as yet uncharacterised signalling pathways. PEDF also regulates inflammatory signalling to reduce PC growth and metastasis by inhibiting caveolin-1-induced activation of interleukin (Il)-8 expression, a protein overexpressed in PC and associated with advanced disease [29]. Il-8 expression in PC is linked to metastasis, development of castration-resistant PC, and chemoresistance, and PEDF may inhibit PC progression by interacting with PEDF-R and regulating PPAR-γ/NF-κB signalling and subsequent Il-8 expression in PC cells [30]. In summary, PEDF is negatively regulated by the pro-inflammatory TME and it may be important for re-directing local immune cells towards an anti-tumour response.

### 4.2. Breast Cancer

Oestrogens and progesterone are critical for post-natal breast development, breast lobule expansion during the luteal phase of the menstrual cycle, and proliferation of milk-producing alveoli during pregnancy [1]. Oestrogen receptor (ER)-α expression by luminal epithelial cells varies throughout the menstrual cycle, whereas progesterone receptor (PR) expression remains constant, with low ER and PR expression observed in normal breast tissue [1]. Oestrogen drives early tumorigenesis of ER+ BC via paracrine mechanisms and later, BC progression via autocrine mechanisms [1]. Different PR isoforms exhibit unbalanced expression during BC progression and a complex crosstalk between ER- and PR-mediated signalling appears to exist in BC [1].

PEDF expression is downregulated in human BC tissue compared to normal breast tissue, with reduced PEDF expression associated with increased tumour microvessel density (MVD), high risk of relapse, and worse survival [44]. Dysregulated lipid signalling associated with obesity appears to downregulate PEDF expression in peritumoral adipose and stromal tissue in locally advanced BC, which may promote tumour growth and progression [45] and could be the mechanism underlying the increased risk of developing BC in obese postmenopausal women. Oestradiol may drive endocrine resistance in ER+ BC tissue by downregulating PEDF and upregulating ER-α expression [22]. This study showed that PEDF may mitigate tamoxifen resistance and reduce BC proliferation by reducing ER-α, protein kinase B (Akt), and receptor tyrosine kinase rearranged during transfection (RET) expression. This indicates a possible link between oestrogen signalling and PEDF in BC, with potential implications for improving our understanding of BC development during menopause.

Epithelial-mesenchymal transition (EMT) describes a process critical for tumorigenesis and metastasis whereby epithelial cells acquire a genetic profile that facilitates migration and invasiveness [46]. PEDF may prevent BC cell EMT by regulating nuclear factor-kappa B (NF-κB) and extracellular signal regulated kinase (ERK)/Akt signalling to decrease the expression of EMT markers vimentin, Snail, fibronectin, and matrix metalloproteinases (MMPs), and increase E-cadherin expression, an important cell adhesion molecule [25,26]. The ability of PEDF to potently inhibit tumour angiogenesis is another important anti-tumour mechanism, whereby PEDF reduces BC growth and improves survival potentially by downregulating pro-angiogenic hypoxia inducible factor (HIF)-α/α-smooth muscle actin signalling in BC cells [24]. These findings show decreased PEDF expression promotes a pro-angiogenic, pro-metastatic tumour microenvironment (TME) essential for facilitating BC growth and progression.

**Figure 2:**
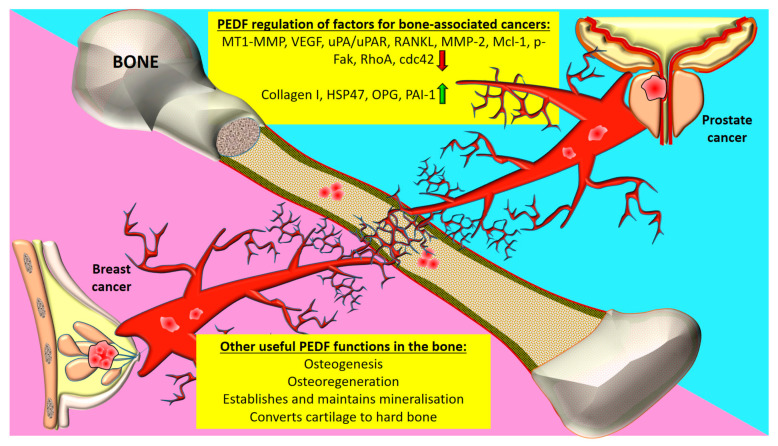
Roles of PEDF in osteotropic tumours and in bone. The metastasis of tumours, such as those in the breast and prostate to bone, requires the activities of such pro-metastatic factors as MT-1-MMP and uPA. Key: dc42, cell division control protein 42 homolog; HSP47, heat shock protein-47; Mcl-1, myeloid cell leukemia 1; MMP, matrix metalloproteinase; MT1-MMP, membrane type-1 metalloproteinase/MMP-14; OPG, osteoprotegerin; PAI-1, plasminogen inhibitor activator-1; VEGF, vascular endothelial growth factor; p-FAK, phosphorylated focal adhesion kinase; RANKL, receptor activator of nuclear factor kappa-Β ligand; RhoA, Ras homolog family member A; red arrow—downregulation; green arrow—upregulation; red markers in bone—tumour cells.

Loss of PEDF expression is implicated in BC metastasis, with SERPINF1 gene expression downregulated in clinical brain metastases compared to primary BC [47]. PEDF promotes BC cell apoptosis in metastatic lesions by activating Fas, Max, peroxisome proliferator-activated receptor-gamma (PPAR-γ), and caspase-2 [17]. PEDF also elicits anti-metastatic function by preventing activation of focal adhesion kinase (FAK) blocking the pro-invasion proteolytic action of MMP-14 (membrane type-1 metalloproteinase/MT1-MMP) and urokinase plasminogen activator receptor, uPAR [27]. MMP-14 mediates tumour invasion and angiogenesis and PEDF may reduce tumour cell metastatic potential by negatively regulating MMP-14, and subsequent MMP-2 activation in various cancer types, including BC [48]. These factors are most relevant to bone metastases from cancers such as BC and PC (Figure 2). In bone and bone-associated cancers, PEDF has been shown to regulate the expression components of bone extracellular matrix including collagen-I and heat shock protein-47 (HSP47) [49], as well as other factors associated with cancer cell metastasis including osteoprotegerin, receptor activator of nuclear factor kappa-Β ligand (RANKL), plasminogen inhibitor activator (PAI)-1, vascular endothelial growth factor (VEGF), Ras homolog family member A (RhoA), cell division control protein 42 homolog (cdc42), and myeloid cell leukemia 1 (Mcl-1) [50,51,52]. The impact of sex hormones or menopausal status on the regulatory role of PEDF on these tumorigenic molecules in sex hormone-responsive cancers and metastasis remains to be investigated. These studies indicate that PEDF regulates genomic pathways which alter the expression of adhesion molecules and proteases, ultimately inhibiting anchorage-independent growth and invasion of BC cells into surrounding tissues or vessels at both the primary and secondary tumour sites. Oestrogen regulates PEDF in the endometrium [20], ovarian surface epithelial cells [18] and granulosa cells [19]. There has only been one report indicating that PEDF expression is under the regulatory control of oestrogen in BC cells [24], but this study did not utilise a microenvironment containing other forms of oestrogen-E1 and E1S.

### 4.3. Female Reproductive Tract Cancers

#### 4.3.1. Ovarian Cancer

During ovarian folliculogenesis, conversion of androgens to E2 by aromatase-expressing granulosa cells contributes to local and systemic oestrogen levels, regulates the secretion of pituitary-derived sex hormones, and promotes uterine angiogenesis by increasing VEGF and angiotensin-I expression [53]. E2 appears to play an important role in promoting tumour growth and chemoresistance in ovarian cancer (OC), with the ratio of ER-α/ER-β increasing during OC progression compared to normal ovarian tissue [4]. Studies indicate that ER-α-mediated signalling is associated with pro-tumour effects, while ER-β-mediated expression and signalling is associated with anti-tumour effects and improved patient outcomes [4].

PEDF expression is reduced in OC tissue compared to normal tissue and is negatively regulated by E2 in ER+ OC cells via an oestrogen response element identified within the PEDF promoter [18]. Loss of PEDF expression causes oxidative damage in the ovary, manifesting as reactive oxygen species accumulation and activation of NF-E2-related factor-2 (Nrf2) signalling [54]. Oxidative stress is strongly associated with the OC microenvironment and is implicated in ovarian tumorigenesis, metastasis, and chemoresistance, which may be linked to Nrf2 activation [55]. Recently, endogenous PEDF present in malignant ascites was identified as the protein responsible for reducing OC cell proliferation and tumour growth, although the cellular source of PEDF in ascites is yet to be confirmed [56]. Research to date implicates PEDF loss and oxidative stress in OC progression and identifies oestrogen as a negative regulator of PEDF expression. Further research is needed to define the molecular mechanisms involved in PEDF action in OC.

#### 4.3.2. Endometrial Cancer

ER-α, ER-β, and AR are expressed throughout the endometrium, with cell-specific expression changing throughout the menstrual cycle [57]. Endometrial proliferation during ovulation is tightly regulated by E2 signalling via ER-α, with local conversion of circulating androgens, with E1S also regulating the endometrium [57]. Oestrogens are key drivers of endometrial hyperplasia and type I endometrial cancer (EC), with key enzymes involved in converting oestrogen precursors to bioactive E2, such as aromatase, steroid sulfatase, and 17 β-hydroxysteroid dehydrogenase type 1, also overexpressed in EC [57]. E2 signalling via ER-α, a variant ER isoform, is thought to promote cell cycle progression in EC cells (ECCs) by activating ERK signalling pathways to upregulate cyclin D1 [4].

PEDF and PEDF-R are expressed in normal and cancerous endometrium [20], with loss of PEDF expression associated with endometrial hyperplasia, a precursor for EC [23]. E2 and progesterone differentially regulate PEDF secretion by endometrial epithelial cells, ECCs, and tumour-resident fibroblasts, with reduced PEDF associated with increased ECC proliferation [23]. Tamoxifen is an aromatase inhibitor used to treat ER+ BC, which may cause endometrial hyperplasia in post-menopausal women by downregulating PEDF and increasing ER-α and ER-β expression over time [58]. PEDF may protect against tamoxifen-induced endometrial hyperplasia and EC by reducing ER-α expression, preventing VEGF-mediated angiogenesis, inhibiting pro-survival signalling, and increasing pro-apoptotic signalling in the uterus [58]. PEDF may also protect against endometrial tumorigenesis and metastasis by promoting ECC apoptosis and reducing invasiveness by downregulating MMP-9 and VEGF [59]. To summarise, sex hormones regulate endometrial PEDF expression and PEDF protects against oestrogen-driven pathologies in the uterus. PEDF antagonises sex hormone-induced tumour growth and endocrine resistance in EC, potentially by downregulating ER-α.

## 5. Conclusions and Future Perspectives

It is expected that the potential prognostic role of PEDF across a diverse range of cancers may be better defined with larger studies focused on specific tumour types, with larger patient numbers and patient outcome data being available. Current knowledge indicates that PEDF is regulated by sex hormones and plays important direct and indirect anti-tumour roles in cancers arising in sex hormone-responsive tissues. PEDF plays an important inhibitory role in tumour angiogenesis as well as negatively regulating the proliferation, survival, and invasiveness of sex hormone-responsive tumour cells. Furthermore, PEDF has demonstrable efficacy in reducing tumour growth, metastasis, and treatment resistance in sex hormone-responsive tumours, which may be due to PEDF interactions with inflammatory and sex hormone signalling pathways in tumour cells. Across a wide range of cancers, PEDF has been shown to be efficacious as an adjuvant therapy in preclinical studies in sex hormone-responsive cancers [17,33,34], as well as cancers arising in other tissues [60,61,62,63], providing the rationale to consider evaluating this serpin in the clinical setting. There is a complex interplay between sex hormones and the cells and acellular structures of the TME. The hypoxic TME downregulates PEDF and is associated with chronic inflammation and oxidative stress, which drives fibrosis, angiogenesis, tumour growth, and progression. PEDF prevents the development of tumour-associated stroma by decreasing collagen deposition, while also increasing tumour cell adhesion to collagen to reduce growth and metastasis of sex hormone-responsive tumours. Considering the apparent link between oestrogens and PEDF, and between oestrogen and collagen in normal tissue, a potential link between sex hormones, PEDF and tumour fibrosis may also exist, and warrants further investigation.

The immunosuppressive effects of oestrogen within the TME facilitates tumour immune evasion and reduces PEDF expression. As such, PEDF may counteract sex hormone-mediated tumour immune evasion to promote an anti-tumour immune response. Further research is required to clarify the molecular mechanisms and clinical impact of sex hormones on PEDF in tumorigenesis, metastasis, progression, and treatment resistance. A current lack of clinically relevant animal models recapitulating the complex human hormonal milieu limits the clinical development of PEDF as an anti-cancer treatment for sex hormone-responsive tumours. Given the importance of sex hormones in cancer development and their regulatory role over PEDF, future studies should attempt to develop in vitro and in vivo models representing physiologically relevant systemic sex hormone levels, which could then be used as investigational tools to validate the therapeutic potential of targeting PEDF.

## Figures and Tables

**Table 1:** Summary of known role of sex hormones on pigment epithelium-derived factor (PEDF) expression.

Tissue	Cell Type	PEDF Role in Tissue	Sex Hormones and PEDF Expression	Study Type	References
Breast	BC cells	Blocks ERE signalling in BC and may prevent endocrine treatment resistance.	E2 ↓↓ PEDF	In vitro	[22]
Ovary	OSE cells	Regulates cell growth by blocking E2-mediated proliferation.	E2 ↓↓ PEDF	In vitro	[18]
	OC cells				
	GCs	Regulates follicular angiogenesis.	E2 ↓↓ PEDF	In vitro	[19]
			HCG ↓↓ PEDF		
			P4 ↓↓ PEDF		
Endometrium	ECCs	Regulates endometrial angiogenesis during reproductive cycles.	E2 ↓↓ PEDF	In vitro	[20]
				In vivo	
				Clinical	
	PPECs		P4 ↑↑ PEDF		
	ESFs	Inhibits proliferation of normal and malignant endometrium.	E2 ↓↓ PEDF	In vitro	[23]
	EECs				
	ECCs		P4 ↑↑ PEDF	In vivo	
Prostate	PSCs	Prevents stromal vasculature and epithelial tissue growth.	DHT ↓↓ PEDF	In vitro	[21]

Key: BC: breast cancer; DHT: dihydrotestosterone; E2: oestradiol; ECCs: endometrial cancer cells; EECs: endometrial epithelial cells; ERE: oestrogen response element; ESFs: endometrial stromal fibroblasts; GCs: granulosa cells; HCG: human chorionic gonadotrophin; OC: ovarian cancer; OSE: ovarian surface epithelial; P4: progesterone; PPECs: proliferative phase endometrial cells; PSCs: prostate stromal cells; ↓↓: downregulate; ↑↑: upregulate.

**Table 2:** Summary of anti-tumour effects of PEDF in sex hormone-responsive tissues.

Tumour Type	PEDF Anti-Tumour Role	Pathways Involved	Effectors	References
Breast	Anti-proliferative ↓↓ Chemoresistance	RET/Akt	ER-α	[22]
	Anti-angiogenic	HIF-1α/α-SMA	Unknown	[24]
	↓↓ Invasiveness	ERK/Akt, NF-κB, FAK	E-cadherin, MMP-2, MMP-9, MMP-14, vimentin, Snail, fibronectin, uPAR	[25,26,27]
	Pro-apoptotic	PPAR-γ	Fas, MAX, caspases	[17]
Prostate	Anti-metastatic	PAI-2, Caveolin-1, PPAR-γ/NF- κB	uPA, proteases, Il-8	[28,29,30]
	Anti-angiogenic			
	Anti-proliferative			
	Pro-apoptotic	LR/JNK/PPAR-γ/FasL	Caspases	[31,32]
	↑↑ Chemosensitivity	Il-8/CXCR1	Macrophages	[33,34]
	↓↓ Invasiveness			
	Anti-proliferative			
	↑↑ Anti-tumour immune response	Unknown	PEDF-R, CD-47, ATP5B	[35]
Ovarian	Anti-proliferative	Unknown	Unknown	[18]
	Pro-apoptotic			
Endometrial	Pro-apoptotic	PI3K/Akt/mTOR, JNK	VEGF, MMPs, ER-α, c-Myc	[23]
	Anti-metastatic			
	Anti-angiogenic			

Key: α-SMA: α-smooth muscle actin; Akt: protein kinase B; ATP5B: ATP synthase F1 subunit beta; CXCR1: C-X-C motif receptor 1; ER: oestrogen receptor; ERK: extracellular signal regulated kinase; FAK: focal adhesion kinase; FasL: Fas ligand; HIF: hypoxia inducible factor; Il: interleukin; JNK: c-Jun N-terminal kinase; LR; laminin receptor; MMP: matrix metalloproteinase; mTOR: mammalian target of rapamycin; NF-κB: nuclear factor-kappa B; PI3K: phosphoinositide 3-kinases; PAI-2: plasminogen activator inhibitor-2; PPAR-γ: peroxisome proliferator-activated receptor-gamma; PEDF-R: PEDF receptor; RET: receptor tyrosine kinase rearranged during transfection; uPA: urokinase plasminogen activator; uPAR: uPA receptor; VEGF: vascular endothelial growth factor; ↑↑: increases; ↓↓: decreases.

## Abbreviations

*Akt*	Protein Kinase B
*AR*	Androgen Receptor
*ATP5B*	ATP Synthase F1 Subunit Beta
*Cdc42*	Cell Division Control Protein 42 Homolog
*CRPC*	Castration-Resistant PC
*CXCR1*	C-X-C Motif Receptor 1
*DHT*	Dihydrotestosterone
*EC*	Endometrial Cancer
*ECC*	EC Cell
*EMT*	Epithelial-Mesenchymal Transition
*ER*	Oestrogen Receptor
*ERK*	Extracellular Signal-Regulated Kinase
*FAK*	Focal Adhesion Kinase
*FasL*	Fas Ligand
*HIF*	Hypoxia-Inducible Factor
*HSP47*	Heat Shock Protein-47
*Il*	Interleukin
*JNK*	c-Jun N-Terminal Kinase
*MAPK*	Mitogen-Activated Protein Kinase
*Mcl-1*	Myeloid Cell Leukemia 1
*MMP*	Matrix Metalloproteinase
*mTOR*	Mammalian Target of Rapamycin
*MVD*	Microvessel Density
*NF-κB*	Nuclear Factor-Kappa B
*Nrf2*	NF-E2-Related Factor-2
*OC*	Ovarian Cancer
*PAI*	Plasminogen Inhibitor Activator
*PC*	Prostate Cancer
*PEDF*	Pigment Epithelium-Derived Factor
*PI3K*	Phosphoinositide 3-Kinase
*PKC*	Protein Kinase C
*PPAR-γ*	Peroxisome Proliferator-Activated Receptor-Gamma
*PR*	Progesterone Receptor
*RET*	Receptor Tyrosine Kinase Rearranged During Transfection
*RhoA*	Ras Homolog Family Member A
*T*	Testosterone
*TME*	Tumour Microenvironment
*TNBC*	Triple Negative Breast Cancer
*TNF*	Tumour Necrosis Factor
*uPA*	Urokinase Plasminogen Activator
*uPAR*	uPA Receptor
*VEGF*	Vascular Endothelial Growth Factor
